# Radical Healing Approaches For Black Maternal Health: A Critical Review

**DOI:** 10.1007/s40615-025-02639-8

**Published:** 2025-09-26

**Authors:** Haley M. Cooper, Jonisha R. Brown, Virginia Gil-Rivas

**Affiliations:** 1 Health Psychology Program, University of North Carolina at Charlotte, University City Blvd. Colvard 3027D, Charlotte, NC 28223 USA; 2 Family Medicine, Wake Forest School of Medicine, Charlotte, NC USA; 3 Department of Psychological Sciences, University of North Carolina at Charlotte, Charlotte, NC USA

**Keywords:** Maternal health, Radical healing, Intersectionality, Gendered racism, Strengths-based, Empowerment

## Abstract

Black birthing people are intrinsically valuable individuals, as well as members of families, organizations, communities, and society. Yet, gendered racism exposes them to unique sources of stress that can accelerate physiological aging, increase risk for adverse health conditions throughout the lifespan, and contribute to preventable morbidity and mortality risk during pregnancy, delivery, and the postpartum period. Existing research focuses on *coping* strategies Black birthing people can employ to alleviate the effects of stress from gendered racism. While efforts to provide immediate stress relief are essential, interventions to reduce gendered racism in Black birthing people’s environments and prevent future stress exposures are necessary to eliminate persistent and severe maternal health disparities. This critical narrative literature review applies a psychological framework of radical healing (French et al., 2020) to explore the relationship between *resistance* strategies and factors associated with maternal health. Specifically, this paper synthesizes evidence about strategies to empower Black birthing people and their communities with strength to resist gendered racism at internalized, interpersonal, and institutional levels. It also explores opportunities for organizations and institutions to combat gendered racism and promote equitable maternal health outcomes. Implications for future research, practice, and policy are discussed.

## Defining the Black Maternal Health Crisis

Black birthing people (BBP)—people with the physiologic capacity for pregnancy who identify as Black [1]—are intrinsically valuable individuals and members of families, organizations, communities, and society. Pregnancy, childbirth, and the postpartum period can be a joy-filled and transformative time for BBP and their families. Deaths during this time–80% of which are preventable–can have lasting effects on families and communities who experience these often traumatic and unnatural losses [2]. Unfortunately, BBP in the United States are three times more likely to die from pregnancy-related causes than white birthing people (WBP) [3] and are more likely to die from preventable causes [4]. Racial disparities in maternal mortality have lasted more than 100 years and persist regardless of income or education level [5, 6]. For every one maternal death, there are an estimated 50–100 cases of severe maternal morbidity in the U.S. Severe maternal morbidity includes unexpected outcomes of labor and delivery, such as eclampsia, cardiac arrest, heart failure, and respiratory failure, that can lead to short- and long-term health consequences for birthing persons and infants [5, 7]. Estimates suggest that BBP are twice as likely as WBP to experience severe maternal morbidity [8].

## Gendered Racism, Stress, and Pregnancy Outcomes

Researchers have increasingly applied an intersectional approach [9–11] to examine gendered racism as a root cause of racial disparities in birth outcomes, rather than biological or behavioral differences between BBP and WBP [12]. Gendered racism includes the structures, systems, and ideas that create and perpetuate inequities for members of minoritized race and gender groups [13–15]. Structural gendered racism can expose BBP to chronic stress by limiting access to safe, stable housing and neighborhoods, healthy working conditions with adequate wages, and healthcare benefits such as paid family or sick leave [16, 17]. Even with adequate access to health-related resources, BBP face daily microaggressions due to historically-rooted stereotypes (i.e., Angry Black Woman, Strong Black Woman/Superwoman Schema, Jezebel) [18–20]. Microaggressions are associated with increased stress reactivity [21–23] and may become more frequent for BBP as their socioeconomic position increases [24], a pattern consistent with the racial disparities in birth outcomes that persist across levels of socioeconomic status.

Gendered racial stereotypes can also render BBP invisible, incompetent, and incapable of experiencing pain in the eyes of healthcare systems and providers [15, 17, 25–27]. BBP are more likely to report harsh language, rough handling by hospital personnel, dismissed requests for help, and violations of physical privacy than birthing people from other racial backgrounds [25, 28, 29]. Incidences of obstetric violence (e.g., performance of nonconsensual transvaginal ultrasounds, use of sexualized language during procedures) indicate that for BBP, encounters with perinatal care systems themselves can be sources of traumatic stress [30]. BBP who are transgender men or transmasculine, nonbinary, gender nonconforming and/or genderqueer also face unique barriers to equitable, quality healthcare. For example, Black transgender and gender expansive individuals (TGE) have reported experiencing identity-based exclusion from sexual and reproductive healthcare services and inadequate provider knowledge of transgender health [31–36]. These challenges can potentially expose Black TGE, who share experiences of racist oppression and adverse social determinants of health with cisgender Black women [31], to greater vulnerability of obstetric violence and maternal mortality and morbidity.

Taken together, evidence suggests that gendered racism restricts access to health-related resources, subjects BBP to daily microaggressions, fuels discrimination in healthcare, and increases exposure to chronic stress that can produce allostatic load. Allostatic load can increase the risk of chronic conditions including cardiovascular diseases and mental health disorders, which are leading causes of morbidity and mortality [2, 37, 38]. BBP demonstrate higher levels of allostatic load and are more likely to enter pregnancy with one or more stress-related health conditions [39–44]. Stress has also been associated with pregnancy complications (i.e., hypertensive disorders of pregnancy, gestational diabetes, preterm birth, and low birthweight) that can increase the risk of cardiovascular disease after childbirth [44–49]. Given the high frequency at which BBP report encountering daily life stressors and gendered racism-related stressors in their daily lives [24, 46, 50], disrupting the cascading effects of gendered racism-related stress on preconception and perinatal health is a key target for interventions to improve outcomes during and beyond the perinatal period.

## Addressing Stress

Research exploring coping mechanisms employed by BBP suggests that religion and spirituality, identity affirmation, social support, and centrality of gendered racial identity are strategies BBP can use to help buffer the effects of stress from gendered racism [51–55]. Cultivating effective coping skills is a vital strategy for managing emotional responses and reaffirming one’s sense of self-worth when threatened by experiences with oppression [51]. However, designing interventions with an exclusive focus on how to appropriately cope with gendered racism will fall short of addressing the root causes of health disparities in this population [51, 56]. Disrupting longstanding disparities in perinatal health requires strategies that not only help BBP navigate gendered racism but also empower them to resist the conditions that expose them to greater health risks. Solutions-focused frameworks rooted in an intersectional analysis of oppression can guide efforts to facilitate healing for BBP by actively challenging the pervasiveness of gendered racism in their daily lives.

## Radical Healing Framework

**Fig. 1:**
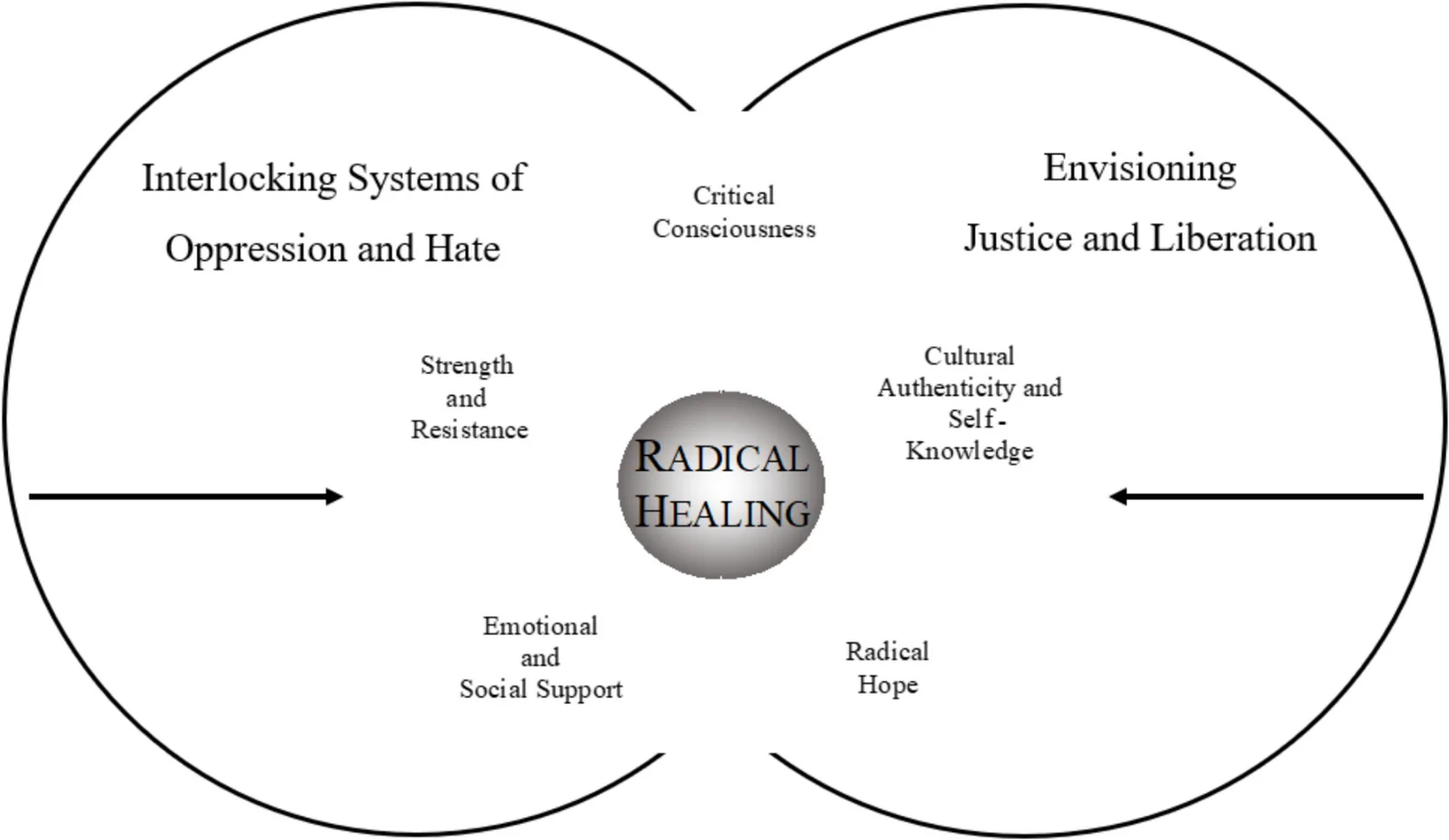
A psychological framework of radical healing [56]

A psychological framework of radical healing (see Fig. 1) provides a foundation for moving beyond reactive, individual-level strategies for coping with oppression and toward proactive, multisystemic approaches to transform oppressive conditions that create health inequities for BBP. The *Psychology of Radical Healing* framework [56] was developed to advance thriving among Black, Indigenous, People of Color (BIPOC) individuals and communities in the context of chronic stress and trauma from racism; this framework can therefore be applied to BBP. While this framework was first published in 2020, it is rooted in foundational concepts from other strengths-based approaches for BIPOC communities, including the psychology of liberation, Black psychology, ethnopolitical psychology, Black Feminism and intersectionality theory [56, 57]. It defines *healing* as the capacity of BIPOC communities to sit within and move between two ends of a healing continuum, with recognition of and resistance against structural injustices at one end and the imagination and pursuit of possibilities for wellness and freedom at the other [56, 57]. Approaches to healing within this framework are considered *radical* in that they address root causes of health compromising conditions [58].

Radical healing consists of five anchors that work in tandem to promote the wellbeing of BIPOC communities. First, radical healing approaches uplift the development of *critical consciousness*–the ability to analyze systems of inequality and situate one’s experiences with oppression within a systemic context [56]. Cultivating this consciousness can help prevent self-blame for oppression and enable acts of resistance [57, 59, 60]. *Strength and resistance* is the second anchor of radical healing, which honors the strength of BIPOC individuals to pursue joy and wellbeing in their own lives and pursue transformative change in their communities [56, 60]. This anchor connects the recognition and resistance against oppression to the visioning of justice and liberation [56]. Demonstrating strength through acts of resistance can also connect BBP to others with similar experiences, bolstering individuals’ and communities’ capacities to respond to future threats of oppression [51, 59].

Third, the anchor of *cultural authenticity and self-knowledge* encourages re-definitions of one’s identity and culture against definitions imposed by oppressors [56, 57]. This anchor contests the dominant belief that Western approaches to health and wellness, many of which are steeped in racist histories, as the most or only legitimate forms of health knowledge and practice. Radical healing approaches honor the return to ancestral healing practices in communities of color. Radical healing also emphasizes the fourth anchor of *collectivism*, which reflects the power and sense of belonging that BIPOC people derive through connection to their community and culture [56]. The framework describes healing as a collective act that connects the wellbeing of individuals to that of their communities and describes collectivism as a means for accessing emotional and social support [61]. Finally, the fifth anchor of *radical hope* centers the courage to envision the possibility of freedom and liberation. Radical hope encompasses the belief in and desire for a better future, grounded in an understanding of and pride in the past [56, 62]. These anchors can be harnessed to inform culturally relevant, solutions-focused research studies and interventions for BIPOC communities, including Black birthing populations.

## Current Study

Research documenting and explaining disparities in maternal mortality and morbidity has been essential for raising awareness of the causes and effects of the maternal mortality crisis among BBP. However, exclusively focusing on disparities and their causes can reinforce powerful deficit discourses which position BBP as problem-prone and in need of fixing [63]. Deficit approaches (i.e., approaches that exclusively identify, describe, and attempt to address problems) can overlook the resources and capacities of BBP, which can serve as foundational strengths upon which health interventions can be built. A psychological framework of radical healing incorporates the strengths and assets of BBP and communities as part of the solution to health inequities and their root causes. While the framework itself is relatively new to the field of psychology, its foundational concepts have decades-long histories across academic disciplines involved in maternal health initiatives. It is unclear to what extent current approaches to research and intervention for Black maternal health are guided by components of the radical healing framework. The current critical literature review investigates the extent to which research identifies opportunities for empowerment of Black birthing persons and synthesizes the evidence for culturally relevant, strengths-based strategies to improve perinatal health outcomes.

## Method

### Approach

Critical reviews involve the application of a theoretical framework, a literature search of diverse sources, and analysis and synthesis of the identified material [64]. Critical reviews enable conceptual innovation through the critical evaluation of key studies on a research topic, without comprehensively including all relevant literature addressing that topic [65]. This approach was selected to provide researchers, practitioners, and affected populations with key information about promising strategies to support Black maternal health and guide the selection of topics for future research and intervention efforts.

### Eligibility Criteria

This narrative review includes published, peer-reviewed studies that identify evidence of strategies to promote radical healing for BBP. Studies were eligible if BBP– Black or African American people of reproductive age (15–49 years old) with the physiologic capacity for pregnancy–were the exclusive population of focus or if they were included in the study sample. Studies were also eligible for review if they focused on promoting perinatal health and wellbeing or preventing morbidity and mortality before pregnancy, during pregnancy and childbirth, or within one year postpartum. Original full text articles written in English of all study designs, except for review articles, were included. Only studies conducted in the United States were eligible for inclusion due to its unique context of anti-Black racism [66]. An initial literature search indicated that research related to Black maternal mortality and morbidity started to surge in 2004. Thus, articles published from 2004 to 2024 were eligible for inclusion.

Strengths-based studies were included for review. In this study, strengths-based research was defined as approaches that (1) center the voices, lived experiences, and capacities of BBP and their communities, and (2) explore ways to build on these assets to inform culturally relevant solutions to promote wellbeing. In addition, articles that included at least one anchor of radical healing were selected for review. The research team compared each study description against the radical healing anchor definitions from French and colleagues [56]. The presence of each anchor was validated through direct mentions of the anchor itself or key components of the anchor’s definition. For articles that did not explicitly identify radical healing concepts or their definitions but did describe themes consistent with the radical healing anchors, group discussions were used to determine inclusion or exclusion.

### Search Strategy

The search was completed in August 2024 using Academic Search Complete, PsycINFO, and PubMed databases. All article fields were searched. The key search terms used were: (black or african american or african-american or black american) AND (maternal health or maternal mortality or maternal deaths or pregnancy related deaths or maternal morbidity) AND (birthing people or women or pregnant women or birthing individuals) AND (resistance or resist or empowerment or empowering or empower or strengths based or strength or heal or healing or collectivism or critical consciousness or radical hope or cultural authenticity or self-knowledge).

### Selection Process

A team of three reviewers participated in the abstract screening and full-text review process. References identified using the search strategy above were imported into Rayyan, a systematic review software tool. An initial sample of 10 percent of identified abstracts were screened independently by all three reviewers. The first author then facilitated group discussions to assess whether reviewers were applying eligibility criteria in a consistent fashion. These discussions were used to refine the screening process before one reviewer screened the remaining articles. Articles selected for full-text review were independently screened by two reviewers. Any discrepancies during independent reviews were resolved via group discussion, with one reviewer serving as a tiebreaker as needed. Reasons for article exclusion were documented. Example exclusion reasons included wrong study design (e.g., deficit-focused; exclusively documenting or explaining racial disparities), wrong outcome (e.g., examining maternal factors to predict infant health or child development outcomes), or wrong population (e.g., assessing physician outcomes from interventions to improve cultural competence in perinatal healthcare).

### Article Assessment

The study design, theoretical frameworks, settings, study participants, perinatal health stages and factors addressed for each selected study were organized into a table to facilitate data analysis. In addition, radical healing anchors were used as deductive codes to analyze the extracted data. Codes were assigned to excerpts of the article that illustrated anchors from the radical healing framework. The presence of anchors in each article was then documented and described in the data analysis table.

## Results

**Fig. 2:**
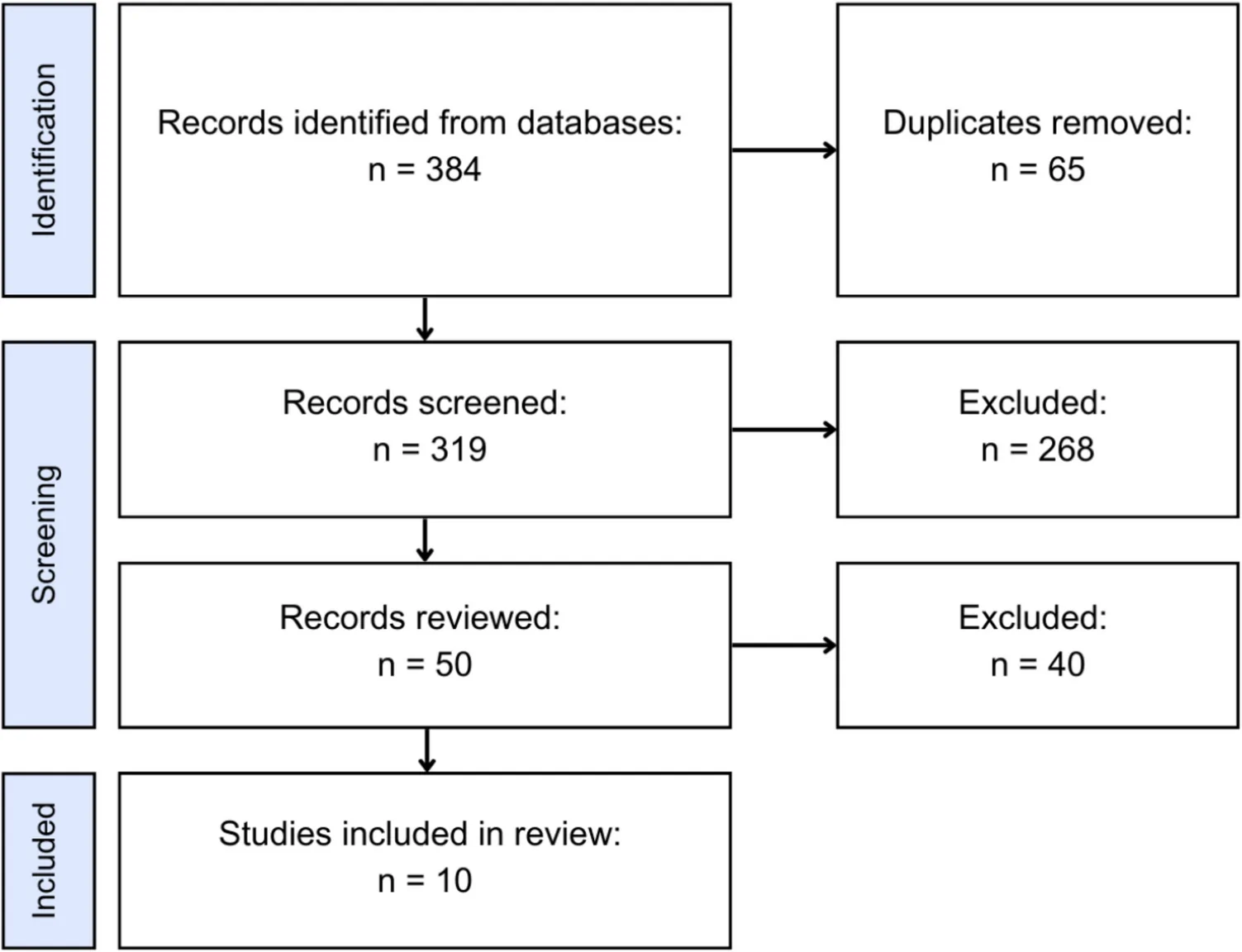
Inclusion flow diagram

The initial search retrieved 319 records for screening after duplicates were removed (see Fig. 2). Strikingly, 97 articles were excluded because they were exclusively deficit-focused. A noteworthy subset of articles (*n* = 24) framed racial health disparities as a result of systemic oppression or used participatory, partnership-based approaches to conduct research with Black birthing communities. Despite this potential for radical healing, these 24 articles ultimately fell short of articulating research questions, methodological approaches, interventions, or interpretations of findings that challenged root causes of maternal health inequities. For example, in a study selected for inclusion in this review, BBP named systemic racism as a barrier to maternal health and uplifted birthing practices outside of the current medical model as a solution [67]. These results informed a text-based intervention to change individual behaviors including nutrition, physical activity, sleep, and healthcare adherence “despite current or past negative experiences” [68]. Early and adequate prenatal care participation is an important determinant of perinatal health outcomes [69]; however, interventions that aim to improve healthcare adherence without aiming to repair past harms or prevent future harms perpetuated by the healthcare system cannot facilitate radical healing for BBP. This is one of several studies that employed strengths-based, participatory methods, but used the results to inform interventions with limited potential for impact on systemic drivers of maternal health.

Other studies framed gendered racism as a cause of racial disparities but employed research methods that reinforced asymmetrical power dynamics and stereotypical representations of BBP. For example, one study used photovoice and appreciative inquiry to engage interdisciplinary maternal and infant health stakeholders during a summit. BBP submitted photos illustrating their daily lives before the summit, during which summit participants reviewed and responded to photos in groups. In their responses, participants made unfounded accusations of sexual abuse toward BBP and appearance-based assumptions about their poverty status [70]. Based on this discussion, participants then made commitments to action to improve maternal and infant health outcomes in their community—without including perspectives from the BBP represented in these images in discussion. The approach used in this study allowed summit participants to project harmful stereotypes onto BBP while excluding BBP from conversations about solutions to improve their own lives.

After excluding ineligible studies, a total of 10 articles were included in the narrative review. The characteristics of included studies are summarized in Table 1. For studies that collected any demographic data (*n* = 5), the samples included BBP from low (*n* = 1) and mixed socioeconomic backgrounds (*n* = 2), primarily in early adulthood (*n* = 3). Seven articles in the review used gender inclusive terms (e.g., birthing person or chestfeeding) or acknowledged the impact of perinatal health disparities on BBP of diverse gender identities. However, no Black TGE individuals were sampled in the selected studies. In addition to birthing individuals, studies also contained samples of informal and formal support persons. These included Black women doulas, mental health service providers, and researchers; family dyads and triads (i.e., Black maternal and grandmother figures); prenatal care providers including midwives and physicians; and program staff. Eight studies included BBP themselves, while two studies exclusively sampled maternal health professionals who were Black women. Studies explored various outcomes across the perinatal period, such as breastfeeding and chest feeding, maternal mental health and stress, healthcare and childbirth experiences, and overall perinatal health and maternal wellness.

**Table 1:** An overview of the included studies

First author and year of publication	Study design and data collection	Theoretical framework(s)	Study population and study setting	Sample size	Stage(s) in perinatal period	Perinatal health outcomes addressed
Duncan et al. (2022). Breastfeeding awareness and empowerment (BAE): A Black women-led approach to promoting a multigenerational culture of health	A mixed-method descriptive study using group process observations and cross-sectional participant questionnaires	Reproductive Justice	Black birthing persons participating in a community-based intervention in greater Philadelphia, Pennsylvania	32 survey respondents and 4 program sessions observed	Pregnancy and postpartum	Breastfeeding initiation, breastfeeding continuation, and perinatal mental health support
Gregorio & Mantri (2024). Portals to the past and bridges to the future: exploring the impact of doulas on the birthing experiences of black and Latinx women	A thematic analysis of popular media sources using grounded theory	None	Blogs, magazine articles, podcasts, and video interviews containing direct accounts from birthing persons and doulas of color	27 direct accounts	Perinatal period with an emphasis on childbirth	Impact of doulas on the perinatal health period and childbirth experience
Hunte et al. (2022). "Black nurses in the home is working": Advocacy, naming, and processing racism to improve Black maternal and infant health	A cross-sectional, qualitative exploratory study using focus groups	Black Feminism and Reproductive Justice	Black pregnant women and program staff (community health workers, nurses, and an office manager) in a home visiting intervention in Portland, Oregon	16 Black birthing program clients and 7 program staff	Pregnancy through 18 months postpartum	Impacts of racism-related stress on Black women's health, pregnancy and parenting; how the program affects this relationship
Matthews et al. (2021). Pathways to equitable and antiracist maternal mental health care: Insights from Black women stakeholders	A cross-sectional, qualitative descriptive study using grounded theory and involving virtual semi-structured interviews	Reproductive Justice and Birth Equity	Black women with expertise in perinatal mental health for Black birthing people, including social workers, birth workers, psychologists, therapists, and researchers	10 Black women perinatal mental health stakeholders	Perinatal period	Maternal mental health
McFarlane et al. (2024). Reframing the experience of childbirth: Black doula communication strategies and client responses during delivery hospitalization	A prospective, qualitative exploratory study using thematic analysis of diary entries and semi-structured interviews	Agency-Identity Model	Black doulas who recently supported a Black birthing person's hospital birth in large cities across the United States	9 Black doulas	Childbirth	Agency in childbirth decision making
Mollard et al. (2024). Reclaiming narratives of empowerment around Black maternal health: a strengths-based, community-informed focus group study	A cross-sectional, qualitative exploratory study involving focus groups	Theory of Maternal Adaptive Capacity	Black females in a Black community health center with sites in Lincoln and Omaha, Nebraska	16 Black females	Not applicable	Not applicable
OjiNjideka Hemphil et al. (2023). Obstetric experiences of young Black mothers: An intersectional perspective	A cross-sectional, qualitative exploratory study involving focus groups	Intersectionality Theory	Young Black pregnant women participating in a community-based prenatal care program in Chicago, Illinois	11 Black pregnant people	Pregnancy	Obstetric care experiences
Robinson et al. (2019). It takes an e-village: Supporting African American mothers in sustaining breastfeeding through Facebook communities	A descriptive, cross-sectional qualitative study using virtual focus groups	Black Feminist Thought and Integrated Model of Behavior Prediction	Black and African American first-time, currently breastfeeding mothers who are members of a Facebook breastfeeding support group	22 Black and African American breastfeeding support group members	Postpartum	Breastfeeding
Rubin & Hines (2023). ‘As long as I got a breath in my body’’: Risk and resistance in Black maternal embodiment	A descriptive, qualitative study using ethnographic interviews conducted over the perinatal period	None	Black pregnant and postpartum women from low-income backgrounds in their homes and attending prenatal care visits in Cleveland, Ohio	17 Black women	Pregnancy and postpartum	Not applicable
Woods Barr et al. (2021). "…[T]this is what we are missing": The value of communicating infant feeding information across three generations of African American women	A cross-sectional, qualitative study using semi-structured interviews	Asset-driven approach guided by Black Feminist Thought and Symbolic Interactionism	Family dyads and triads of African American women—including Black birthing persons, their mother figures, and their grandmother figures—in the southeastern United States	35 African American women, including 15 family dyads and triads	Postpartum	Breastfeeding or chestfeeding

Nine studies used qualitative techniques, including focus groups, interviews, thematic analysis of popular media sources, and ethnography. One study used a mixed-method approach, including observations and a cross-sectional participant survey. Eight of the selected studies used descriptive, exploratory research designs; in these studies, BBP and support persons themselves described anchors of radical healing as factors that have contributed or would contribute to improved perinatal health outcomes.

Two of the selected studies incorporated radical healing anchors as components of community-based interventions to improve perinatal health. In one intervention, BBP and families received comprehensive perinatal services during pregnancy through 18 months postpartum [71]. Example services include home visiting nurses, mental health services, case management and care coordination, and maternal health education delivered by predominantly Black program staff and healthcare providers. This study explored racism-related stress and its effects on health, pregnancy, and parenting among BBP, as well as how the intervention influenced this relationship. Participants reported high satisfaction levels with the program; however, evaluations of the intervention’s effects on perinatal health outcomes addressed by the intervention were not reported. Another intervention used a peer lactation and mental health support group model to facilitate exchanges of knowledge, expertise and support among BBP, facilitators, and experts. This study used self-report survey data to demonstrate high rates of breastfeeding/chest feeding goal achievement and increased community and mental health support. The survey also demonstrated significantly higher rates of breastfeeding initiation and continuation compared to national statistics for BBP [72].

While most selected articles cited theoretical frameworks related to (e.g., Black Feminist Thought) or incorporated in (e.g., Intersectionality Theory) the psychological framework of radical healing, no studies explicitly identified radical healing as a guiding framework. Studies varied in the level of detail provided in how theory guided various stages of the research process, with two studies omitting a theoretical framework altogether. Notably, no articles included radical healing anchors as part of their research questions or examined radical healing anchors as variables of interest. Still, all included studies incorporated anchors from the radical healing framework. Most studies incorporated two (*n* = 5) or three (*n* = 4) radical healing anchors, while only one study reflected all five anchors of the framework. Table 2 summarizes which anchors were incorporated in each article. Detailed descriptions of how each anchor was addressed across selected studies are provided below.

**Table 2:** A summary of radical healing anchors present in selected studies

First author and year of publication	Radical healing anchors present
Duncan et al. (2022). Breastfeeding awareness and empowerment (BAE): A Black women-led approach to promoting a multigenerational culture of health	Collectivism, cultural authenticity and self-knowledge, critical consciousness
Gregorio & Mantri (2024). Portals to the past and bridges to the future: exploring the impact of doulas on the birthing experiences of black and Latinx women	Cultural authenticity and self-knowledge, strength and resistance
Hunte et al. (2022). "Black nurses in the home is working": Advocacy, naming, and processing racism to improve Black maternal and infant health	Collectivism, cultural authenticity and self-knowledge, critical consciousness
Matthews et al. (2021). Pathways to equitable and antiracist maternal mental health care: Insights from Black women stakeholders	Collectivism, cultural authenticity and self-knowledge, critical consciousness
McFarlane et al. (2024). Reframing the experience of childbirth: Black doula communication strategies and client responses during delivery hospitalization	Collectivism, strength and resistance
Mollard et al. (2024). Reclaiming narratives of empowerment around Black maternal health: a strengths-based, community-informed focus group study	Collectivism, cultural authenticity and self-knowledge, critical consciousness, strength and resistance, radical hope
OjiNjideka Hemphil et al. (2023). Obstetric experiences of young Black mothers: An intersectional perspective	Collectivism, strength and resistance
Robinson et al. (2019). It takes an e-village: Supporting African American mothers in sustaining breastfeeding through Facebook communities	Collectivism, cultural authenticity and self-knowledge, strength and resistance
Rubin & Hines (2023). ‘As long as I got a breath in my body’’: Risk and resistance in Black maternal embodiment	Strength and resistance, radical hope
Woods Barr et al. (2021). "…[T]this is what we are missing": The value of communicating infant feeding information across three generations of African American women	Collectivism, cultural authenticity and self-knowledge

### Collectivism

Collectivism was the anchor of radical healing most frequently addressed among the selected articles (*n* = 8). Intervention studies incorporated collectivism through program design, including peer support groups and community events that facilitated shared experiences, mutual support, and encouragement, helping BBP initiate and sustain breastfeeding [71, 72]. These interventions were designed to help BBP strengthen their community support systems. In addition, community spaces created solely for BBP were essential to maternal health and wellbeing. Whether they accessed these spaces through programs, within their own networks, or online support groups, Black birthing community spaces offered BBP an immediate sense of relatability, understanding, and trust [71–73]. Within these spaces, BBP were able to share safely and openly about challenges related to their perinatal health, including stress from racism, and receive validation and support from one another.

In healthcare settings, BBP described how involving a community support system, including family members and doulas, helped balance power differentials between birthing persons and healthcare providers [74, 75]. These collective care experiences enhanced BBP’s ability to understand care options, advocate for their care preferences, and access a sense of safety during their pregnancy and childbirth experiences.

Collectivism was also described as a mechanism for sharing the responsibility of improving maternal health outcomes. For example, mother and grandmother figures shared breastfeeding information and support during the breastfeeding process, which in turn enabled birthing persons to build confidence in their infant feeding practices and facilitated bonding experiences with their family [76]. Having access to supportive family and community ties helped BBP in the selected studies relieve the pressure to remain superhumanly strong or to bear the responsibility of childrearing alone [67, 71]. Both Black women who professionally support this population and BBP themselves identified family and community support as sources of strengths and assets that can be engaged to improve maternal health, including perinatal mental health [77].

### Cultural Authenticity and Self-Knowledge

Anchors of cultural authenticity and self-knowledge were identified in seven of the selected studies. In these studies, BBP recognized how media coverage of maternal mortality suggested that BBP themselves were to blame for racial disparities in maternal health and how positive representations of Black breastfeeding were limited [67, 73]. BBP rejected negative portrayals of the Black birthing experience by redefining Black motherhood as something to celebrate and by joining online communities that uplifted Black breastfeeding. BBP also valued the wisdom and expertise of family elders in communicating breastfeeding information [76].

Interventions to support breastfeeding and perinatal mental health also helped create culturally authentic spaces. Specifically, programs led by Black women staff fostered trust for BBP to receive perinatal health information. These programs also created safe spaces for BBP to process traumatic birth experiences and racism-related stressors [71, 72]. Black women leading these interventions were uniquely positioned to reaffirm BPP’s identities and validate their experiences with racism. In one intervention, program staff encouraged BBP to contribute their own knowledge and experience with perinatal health topics during program sessions, promoting confidence in participants’ self-knowledge related to perinatal health [72]. Additionally, Black women who were stakeholders in perinatal mental health acknowledged spirituality and ancestral practices as essential components of culturally authentic, holistic care for BBP [77]. For example, one marriage and family therapist encouraged their clients to build an altar honoring their Black birthing ancestors and to create space in their daily lives to seek guidance from these ancestors.

Childbirth was a key stage during the perinatal period for radical healing through cultural authenticity. BBP acknowledged the roots of childbirth as a nonmedical, natural experience and expressed a desire to reclaim cultural traditions during their birthing experiences [67, 78]. BBP honored the history of Black birth work, including granny midwives, doulas, and wet nurses, and drew confidence from this history for their own births. Doulas and midwives were viewed as a key connection to an ancestral history of successful childbirth. An analysis of popular media sources indicated that through their roles as advocates, doulas helped BBP regain trust in their bodies to birth successfully and reclaim childbirth as an empowering time [78]. Black maternal health stakeholders also saw doulas and midwives as part of a trusted team to advocate for and support the mental health of BBP [77].

### Strength and Resistance

Six studies described how strength and resistance facilitated radical healing in perinatal health. One theme that emerged from these studies was healthcare settings as a site of resistance [74, 78]. Doulas were vital sources of strength to protect the Black birthing experience within traditional medical systems. For example, doulas of color equipped their Black birthing clients with specific communication tools to resist initiation of unnecessary medical procedures and recommendations that conflicted with their clients’ preferences [74]. In the instance that mistreatment did occur, doulas informed BBP of processes to report abuse, file complaints, and assert their preferences about the presence of specific medical staff (e.g., student doctors). In turn, doulas reported that BBP successfully enacted these skills and tools to preserve their birth plan and their desired cultural traditions. However, BBP’s perceptions and experiences with doula support were not measured in these studies directly.

Family members were also key participants in resistance against medical systems. For example, young BBP reported refusing procedures until their mothers were present because of guidance their mothers provided on questions to ask their providers [75]. They highlighted how their mothers insisted that medical providers slow down and thoroughly explain medical decision making. Doulas also supported spouses and family members to advocate for the birthing person [74]. Together, BBP, doulas, and family members found strength to resist the notion that patients should automatically comply with provider recommendations and traditional decision-making hierarchy in medicine.

Within the context of a maternal mortality crisis and amidst narratives about Black motherhood, BBP described pregnancy and childrearing as empowering acts of resistance themselves [68, 79]. This empowerment was exercised through the vulnerability of sharing challenges and seeking support, a demonstration of resistance against stereotypes that depict BBP as superhumanly strong. Simultaneously, BBP showed strength by taking action to create their ideal birthing experiences and to create the ideal life for their children. BBP who experienced empowerment in their breastfeeding experience also sought to empower others, including by adding new members to online breastfeeding support groups, sharing information with their in-person networks, creating their own support groups, and becoming lactation consultants [73].

### Critical Consciousness

Four of the selected studies incorporated critical consciousness of systemic factors impacting perinatal health. In one study, BBP demonstrated a critical awareness of systemic drivers of maternal mortality disparities and dispelled myths that blame individuals [67]. Interventions designed to improve Black maternal health reflected a critical analysis of structural racism’s effects on health disparities [71, 72]. To address these effects, these interventions included system-level strategies, such as efforts to streamline referral processes in local systems of care and obtain funding for program participants to advance to leadership roles [72]. Mental health providers also recognized cultural, historical, and systemic factors as sources of trauma for BBP and worked with clients to identify the impacts of systemic racism on their mental health [71, 77]. Program staff also provided BBP with information about their rights within various institutions (e.g., housing, education) and advocacy skills to exercise these rights.

### Radical Hope

Only two studies included the anchor of radical hope. In one study designed to identify strengths and elevate potential solutions to improve Black maternal health, BBP articulated the importance of sharing joyful stories about motherhood and highlighting progress in local maternal health initiatives. These strategies were viewed as necessary to counter deficit-focused narratives. BBP also expressed radical hope that they would not be “part of the statistics” [67] and declared that they would have healthy pregnancies and births, even amidst a maternal mortality crisis. In another study, BBP in a racially and economically segregated, chronically stressful environment expressed radical hope for their children’s future [79]. Keenly aware of the impact of structural racism on their lives, BBP in this study strived to maintain their own health to create a better future for their children, free from its effects.

## Discussion

This study extends existing knowledge of maternal health in Black birthing populations by synthesizing strengths-based, culturally relevant research and interventions for BBP’s perinatal health and wellness. The ten articles identified in this review suggest that radical healing in maternal health is facilitated by family members, community networks, and birth workers *collectively* supporting BBP in their efforts to achieve wellness. Radical healing approaches foster *culturally authentic* spaces designed by and for BBP that emphasize connection through shared identities and experiences. Culturally authentic spaces also honor the rich histories of the Black birthing experience in the United States. Radical healing cultivates the *strength* to *resist* obstetric violence and medical mistreatment. Simultaneously, it encourages the *strength* to show vulnerability, ask for and receive support, and *resist* stereotypes of super strength and hyperindependence. Radical healing builds *critical consciousness* of how racism at multiple levels affects perinatal health; it integrates advocacy and systemic change into the design of community interventions. Finally, radical healing inspires *radical hope* for Black birthing joy, wellness, and safe pregnancies and perinatal periods, even within the context of a maternal health crisis.

Although the radical healing framework is relatively new in psychology, the psychological theories (i.e., Black Psychology Liberation Psychology, Ethnopolitical Psychology) integrated within the framework are not. Further, the concept of radical healing was coined as early as 1851 in medicine and introduced as a strategy to transform the medical field in 1999 [57]. Yet, the earliest date of publication that met inclusion criteria was 2019, suggesting that applications of radical healing-related concepts are recent in psychological, public health, and medical research on maternal health. While the ten articles revealed these key insights into radical healing for BBP, none of these studies cited the radical healing framework; one cited Intersectionality Theory, a foundational theory to radical healing. Only one study addressed all anchors of the radical healing framework, while few studies addressed the critical consciousness or radical hope anchors. Most articles used exploratory or descriptive research designs and qualitative research methods. Two intervention studies met the selection criteria, one of which used a mixed-method approach to evaluation.

The lack of articles citing the Radical Healing Framework or theories integrated within it are consistent with previous research suggesting that the inclusion of theory in research on health disparities is limited in general [80]. Health research that rigorously applies theories examining structural, intersectional health disparities and strengths-based solutions for communities of color is even less common and is also in its early stages [80–83]. Indeed, structural barriers to publication, uptake in mainstream academic journals, and use in clinical practice for novel frameworks centering the experiences of BIPOC communities [81, 84] could explain the limited literature identified in this review.

Disrupting pervasive health inequities requires the use of anti-oppression frameworks that challenge the histories of oppression in psychological and medical research, which are maintained in part using “colorblind” theoretical approaches [27, 85–87]. This necessitates exposure among researchers and practitioners to models designed by and for BIPOC communities to guide research and intervention efforts—and reckoning with the systemic disparities in funding and publication processes that currently limit this exposure [81, 88]. Increasing exposure to the framework for radical healing among researchers and practitioners is also key to ensure that all anchors of the framework are addressed in efforts to promote perinatal health.

The radical healing framework asserts that all five anchors work in tandem to promote healing for BIPOC communities [56]. However, only one study in this review included all five anchors of radical healing, which emerged in themes from focus groups with BBP [67]; no studies incorporated all radical healing anchors in their research or intervention designs. Critical consciousness was only examined in four studies in this review. Critical consciousness of systemic oppression is described as a first step for the radical healing process, a precondition for developing hope for a liberated future and strength to resist oppression [56, 60]. Further, only two studies included the anchor of radical hope. This anchor is key to envisioning possibilities for health and wellness free from the effects of oppression, and in turn, inspiring action to pursue these possibilities. Without a focus on cultivating radical hope, efforts aiming to promote radical healing run the risk of exacerbating a sense of disempowerment in Black birthing communities [56]. Engaging fully with anchors of the framework is necessary to facilitate the radical healing process through the dialectic of resisting oppression and moving toward freedom.

The descriptive and exploratory approaches used in the reviewed articles are key to illuminating the intersectional experiences of BBP and underrepresented perspectives in research [82, 89]. However, it is imperative to apply the descriptions of radical healing for maternal health from this body of research to develop and evaluate perinatal health promotion interventions. To date, deficit-focused, individual-level interventions have been the primary strategy used to address the health needs of Black birthing communities [90–93]. These interventions often rely on assumptions that “culture” drives health behaviors and therefore health disparities in BIPOC communities. These assumptions lack the complexity required to tackle interlocking, systemic causes of adverse birth outcomes, and have been ineffective in reducing racial disparities that have existed for more than a century [5, 93, 94]. During the article screening process, we observed that several articles noted systemic barriers to perinatal health as evidence of the need for intervention. However, few studies targeted these identified barriers. While it is important to ensure BBP have the knowledge and skills necessary to engage in perinatal health-promoting behaviors, researchers and practitioners must move beyond an exclusive focus on individual behaviors and toward systemic causes as the primary target of perinatal health intervention efforts. Translating research into action with a radical healing lens offers the potential to transform the root causes of perinatal health disparities and make meaningful changes in the lives of BBP.

Participatory approaches to maternal health research and action are also key to the effectiveness of future radical healing initiatives. BBP and support persons in the selected studies consistently identified gendered racism as a contributor to perinatal health disparities, but most approaches to intervention reviewed during the screening process failed to reflect this insight. This trend highlights a disconnect between researchers and practitioners who plan and implement health interventions and the perspectives of populations impacted by them [95]. Collaborations with BBP, support persons, and community leaders as equitable partners across all stages of intervention development, implementation, and evaluation are essential to ensure that translation efforts honor the voices represented in descriptive research [90, 96–98]. Further, health professionals are largely trained to work *within* systems that create and maintain racial health disparities. Interdisciplinary collaborations with scholars, practitioners and organizers can offer fresh perspectives needed to *reimagine* the design and implementation of health interventions. For example, historians with expertise in medicine, obstetrics, and gynecology can help ensure health innovations do not reproduce past harms; creative professionals can help visualize and verbalize new systems of care.

Notably, seven articles in the review either used gender inclusive terms (e.g., birthing person or chestfeeding), included a statement acknowledging that perinatal health disparities affect BBP of various gender identities, or discussed potential research implications for Black TGE populations. Yet, no studies included samples of Black TGE, compounding the erasure of TGE in perinatal service data [32] and maternal mortality surveillance systems [3] that guide policymaking and resource allocation. To promote safe, healthy pregnancies and perinatal periods for all BBP, it is critically important to go further than simply acknowledging the capacity of Black TGE to become pregnant and move toward their intentional involvement in research and intervention efforts. This work is essential to illuminate perinatal health experiences at the intersection of racism and cissexism that may expose Black TGE to distinct forms of obstetric violence, constrain access to community support, amplify chronic stressors, and exacerbate preconception health disparities [31, 33–35, 99]. Highlighting the strengths of this population, such as the creativity and community-building strategies they employ to resist intersectional oppression, can inform more affirming models of care that promote radical healing [34]. Building an evidence base for Black TGE perinatal health requires strategies to foster trust and support meaningful participation. These include respondent-driven sampling methods, community-engaged research approaches, study leadership by TGE researchers, trauma-informed practices, and assurance of community benefits and compensation commensurate with the risk of retraumatization [100, 101].

The results of this study should be interpreted in the context of key limitations. First, this literature search was not exhaustive; we only analyzed peer-reviewed articles published in academic journals. In a review of five decades of publications on race in psychological research journals, Roberts and colleagues conclude that “the psychological publication process is, understandably, subject to the same structural inequities that stratify the rest of society” [84]. Inequities in the review and publication process, representation on editorial boards, and funding for research projects led by scholars of color, about communities of color, and conducted at the community level have been observed across fields of health research [81, 84, 102–105]. By relying solely on peer-reviewed journals, our search strategy may have inadvertently reproduced these inequities, narrowing the pool of studies and shaping our interpretive lens toward frameworks already deemed “legitimate” to fund and publish within maternal health research and practice. Considering this limitation, future studies should examine grey literature, which often captures knowledge produced by grassroots organizations, activists, and independent researchers producing practice-based evidence for social justice-focused solutions [106, 107]. Utilizing guidelines for searching and synthesizing gray literature can strengthen future reviews that aim to include perspectives in perinatal health that remain underrepresented in peer-reviewed outlets [106].

Secondly, while we employed a structured approach to our search strategy and review process, decision-making to determine whether an article reflected a radical healing approach was subjective. It is possible that other researchers replicating this literature search strategy may consider a different set of articles as consistent with the radical healing framework. Finally, our decision to include diverse study designs resulted in varying levels of methodological rigor across studies. As a result, we were unable to draw consistent conclusions about measurable differences in perinatal health outcomes for BBP associated with radical healing strategies.

Despite these limitations, the findings from this critical review have several implications for maternal health research and practice. First, to combat the contributions of gendered racism within and beyond healthcare to racial disparities in maternal mortality and morbidity, it is critical for researchers to center the radical healing framework given its explicit anti-oppression focus. Measurement development and validation studies to operationalize anchors of the radical healing framework will be an essential next step for quantitative and mixed-methods research in this area. As validated tools develop to measure radical healing constructs, future research should also examine whether components of the radical healing framework are associated with indicators of perinatal health. For example, researchers could assess whether BBP with higher levels of critical consciousness or participation in acts of resistance experience lower levels of gendered racial stress or stress-related health conditions. Longitudinal research examining how the radical healing process unfolds over time can elucidate potential mechanisms and time points for future radical healing interventions to target. Longitudinal studies can also examine the effects of radical healing intervention on perinatal and long-term health outcomes.

Studies that clarify how to translate components of radical healing into practice are also needed to guide future interventions. For example, how might emotional and social support groups for BBP go beyond offering a collective space to cope with oppression–and toward cultivating creativity to reimagine oppressive systems? What does it look like in practice to foster self-knowledge and cultural authenticity as it relates to perinatal health? With the framework’s focus on systemic change as a precondition for wellness among BIPOC communities, future studies should investigate not only how to facilitate radical healing during clinical encounters, but also how changes to perinatal care systems can support these efforts. Emergent models of organizational healing [108] and collective healing [109] uplift potential strategies for organizations and cross-sector collaborations to embed an orientation toward social justice in their policies and practices. These models emphasize the transformative potential of cultivating collective critical consciousness within and across system actors, reimagining organizational hiring and promotion processes to become more inclusive of marginalized voices, measuring success through indicators that prioritize structural change and equity, and fostering opportunities for authentic relationship-building [108, 109]. Efforts to refine, evaluate, and implement models for organizational-level radical healing are essential to inform training for health professionals and staff in community-based organizations, guide delivery of transformative services, and develop tools to assess radical healing in organizations and systems.

The predominance of deficit-focused articles retrieved in our search highlights the need to adopt strengths-based approaches in maternal health research. Despite the unacceptable rates of pregnancy-related deaths in Black birthing populations, most BBP survive pregnancy without complications that harm the birthing person or baby [110]. Research including samples of BBP with positive perinatal experiences and outcomes can produce important insights into protective factors for this population, including the potential role of radical healing processes in facilitating safe and healthy pregnancies. Additionally, research must examine the strengths, needs, and assets within Black TGE populations to ensure the effectiveness of future interventions.

Encounters with obstetric care were sources of fear and sites of gendered racism experiences for participants in the reviewed articles [71, 74, 75, 78]. This finding highlights the need to apply the transformative potential of radical healing within clinical settings so that BBP can access the safe, respectful maternity care practices they deserve. Structural competency and structural humility frameworks in healthcare can offer tools for providers to develop their own critical consciousness of how oppressive structures affect health in Black birthing populations and resist stereotype-driven explanations for health behaviors and outcomes among BBP [111, 112]. Training for healthcare providers should emphasize consciousness of how the authoritative knowledge and hierarchical decision-making structures that typify healthcare encounters can reinforce the oppressive conditions that engender health disparities. Providers should learn about the history of Black birth work, respect the legitimacy of doula and midwifery care, and strive for collaborative approaches to care and decision-making with BBP and all support persons to transform the power dynamics in perinatal care.

Fostering equitable collaborations with community-based organizations is another key strategy for healthcare organizations to build capacity to address structural barriers to health. Community-based organizations often provide resources and services to address social determinants of health that affect outcomes within healthcare systems [113]. Trusting relationships with community members can also enable community-based organizations to reach BBP who are not connected with the healthcare system and provide key insights to healthcare organizations to better support this population [114]. Consistent with previous research that has highlighted preferences for and potential health benefits of race- and gender-concordant healthcare providers among BBP [115–117], Black women-led programs and interventions were trusted and essential sources of radical healing for BBP in the selected studies. Investing in Black women-led community care models is a crucial strategy for addressing racial perinatal health disparities [27, 77, 118, 119]. Considering that many Black birth workers have lived experience with gendered racism and traumatic birth [119], ensuring access to sufficient resources and support is key to the sustainability of these initiatives.

Community-based maternal health organizations are well-positioned to implement radical healing interventions for BBP. These organizations can offer programs to develop advocacy skills among BBP and their support to resist oppression in healthcare settings, a valuable resource for radical healing. Community-based organizations can also offer welcoming community support spaces that foster vulnerability among BBP to share and receive support, enable safety to process past experiences of gendered racism and prevent future ones, and celebrate Black birthing wellness and positive birth stories. Together, these areas for intervention can facilitate radical healing in Black birthing communities.

We would be remiss if we did not acknowledge how executive orders announced by the current administration to eliminate federal support for research related to diversity, equity, and inclusion have ignited a hostile climate for the kinds of research and intervention approaches we are proposing. However, these mandates come at a time where sweeping executive actions to restrict reproductive and gender-affirming healthcare access have escalated threats to the wellbeing of Black birthing populations. Researchers must investigate the consequences of the current health-compromising policy climate to continue highlighting systemic drivers of racial health disparities and building an evidence base for advocacy efforts that advance birth equity. Researchers and practitioners must also remain creatively committed to funding, conducting, disseminating, and collaborating on projects that promote equitable health outcomes. For example, researchers may need to rely more on foundations who are supportive of health equity initiatives than state and federal funding sources for future projects. Identifying outlets for publication that have renewed their dedication to achieving social justice aims will help ensure dissemination of critically important research and evaluation findings.

## Conclusion

Traditionally, maternal health research has amplified the riskiness of pregnancy and childbirth for BBP and informed individual-level interventions to address this population’s perceived deficits. These approaches can lead researchers and practitioners to expect that BBP will experience adverse birth outcomes; when interventions devoid of a multi-level framing fall short of reducing disparities, they can reinforce the narrative that BBP themselves are to blame [43, 120]. Strengths-based approaches are necessary to broaden and balance the portrayal of the Black birthing experience by elevating not only definitions of the maternal mortality problem, but solutions for perinatal health and wellbeing. Culturally relevant strategies that are grounded in Black birthing communities’ lived experiences and responsive to the systemic health barriers are key to promoting perinatal wellness in this population. Radical healing approaches integrate these strengths-based, culturally relevant strategies to build critical consciousness of gendered racism, uplift community-level strengths to resist oppression, foster culturally authentic spaces for connection and support, honor ancestral birthing practices, and cultivate radical hope for positive pregnancy outcomes. By embedding community-driven and resistance-oriented approaches into research and practice, maternal health initiatives can move beyond mitigating disparities and toward fostering Black birthing joy, safety, and wellness.

## Data Availability

This declaration is not applicable.

## Code Availability

This declaration is not applicable.

## References

[1] Julian Z, Robles D, Whetstone S, Perritt JB, Jackson AV, Hardeman RR, et al. Community-informed models of perinatal and reproductive health services provision: a justice-centered paradigm toward equity among Black birthing communities. Semin Perinatol. 2020;44:151267. 10.1016/j.semperi.2020.151267 32684310

[2] Centers for Disease Control and Prevention. Pregnancy-related deaths: Data from Maternal Mortality Review Committees in 36 U.S. states, 2017–2019 [Internet]. Matern. Mortal. Prev. 2024 [cited 2024 Jun 27]. Available from: https://www.cdc.gov/maternal-mortality/php/data-research/mmrc-2017-2019.html

[3] Centers for Disease Control and Prevention. Pregnancy Mortality Surveillance System [Internet]. Matern. Mortal. Prev. 2024 [cited 2024 Jun 27]. Available from: https://www.cdc.gov/maternal-mortality/php/pregnancy-mortality-surveillance/index.html

[4] Louis JM, Menard MK, Gee RE. Racial and ethnic disparities in maternal morbidity and mortality. Obstet Gynecol. 2015;125:690–4. 10.1097/AOG.0000000000000704 25730234

[5] Declercq E, Zephyrin LC. Maternal mortality in the United States: a primer [Internet]. Commonw. Fund. 2020 [cited 2024 Jun 27]. Available from: https://www.commonwealthfund.org/publications/issue-brief-report/2020/dec/maternal-mortality-united-states-primer

[6] Kennedy-Moulton K, Miller S, Persson P, Rossin-Slater M, Wherry L, Aldana G. Maternal and infant health inequality: new evidence from linked administrative data [Internet]. National Bureau of Economic Research; 2022 [cited 2024 Apr 19]. Available from: https://www.nber.org/papers/w30693

[7] Kirby RS, Verbiest S. Kotch’s maternal and child health: problems, programs, and policy in public health. Jones & Bartlett Learning; 2021.

[8] Creanga AA, Bateman BT, Kuklina EV, Callaghan WM. Racial and ethnic disparities in severe maternal morbidity: a multistate analysis, 2008–2010. Am J Obstet Gynecol. 2014;210:435.e1-435.e8. 10.1016/j.ajog.2013.11.039 24295922

[9] Collins PH. Learning from the outsider within: the sociological significance of Black Feminist Thought. Sociol Probl. 1986;33:14–32.

[10] Crenshaw K. Mapping the margins: intersectionality, identity politics, and violence against women of color. Stanford Law Rev. 1991;43:1241–99.

[11] The Combahee River Collective. The Combahee River Collective statement [Internet]. 1977 [cited 2025 Mar 11]. Available from: https://americanstudies.yale.edu/sites/default/files/files/Keyword%20Coalition_Readings.pdf

[12] Crear-Perry J, Correa-de-Araujo R, Lewis Johnson T, McLemore MR, Neilson E, Wallace M. Social and structural determinants of health inequities in maternal health. J Womens Health. 2021;30:230–5. 10.1089/jwh.2020.8882 PMC8020519 33181043

[13] Battle S, Carty D. Gendered racism among women of color [Internet]. 2022 [cited 2024 Sep 27]. Available from: https://blogs.cdc.gov/healthequity/2022/10/06/gendered-racism-among-women-of-color/

[14] Essed P. Understanding everyday racism: an interdisciplinary theory [Internet]. Thousand Oaks: SAGE Publications, Inc.; 1991 [cited 2024 Apr 18]. Available from: https://sk.sagepub.com/books/understanding-everyday-racism-an-interdisciplinary-theory/n2.xml

[15] Laster Pirtle WN, Wright T. Structural gendered racism revealed in pandemic times: intersectional approaches to understanding race and gender health inequities in covid-19. Gend Soc. 2021;35:168–79.

[16] Noursi S, Saluja B, Richey L. Using the ecological systems theory to understand Black/White disparities in maternal morbidity and mortality in the United States. J Racial Ethn Health Disparities. 2021;8:661–9. 10.1007/s40615-020-00825-4 32720294

[17] Tyler E. Black mothers matter: the social, political and legal determinants of Black maternal health across the lifespan. J Health Care Law Policy. 2022;25:49.

[18] Lewis JA, Neville HA. Construction and initial validation of the gendered racial microaggressions scale for Black women. J Couns Psychol. 2015;62(2):289–302. 10.1037/cou0000062 25867696

[19] Rosenthal L, Lobel M. Explaining racial disparities in adverse birth outcomes: unique sources of stress for Black American women. Soc Sci Med. 1982;2011(72):977–83. 10.1016/j.socscimed.2011.01.013 21345565

[20] Woods-Giscombé CL. Superwoman schema: African American women’s views on stress, strength, and health. Qual Health Res. 2010;20:668–83. 10.1177/1049732310361892 PMC3072704 20154298

[21] Clark R, Anderson NB, Clark VR, Williams DR. Racism as a stressor for African Americans: a biopsychosocial model. Am Psychol. 1999;54:805–16. 10.1037//0003-066x.54.10.805 10540593

[22] Lewis JA, Williams MG, Peppers EJ, Gadson CA. Applying intersectionality to explore the relations between gendered racism and health among Black women: Journal of Counseling Psychology. J Couns Psychol. 2017;64:475–86. 10.1037/cou0000231 29048194

[23] Pascoe EA, Smart Richman L. Perceived discrimination and health: a meta-analytic review. Psychol Bull. 2009;135:531–54. 10.1037/a0016059 PMC2747726 19586161

[24] Kwate NOA, Goodman MS. Racism at the intersections: gender and socioeconomic differences in the experience of racism among African Americans. Am J Orthopsychiatry. 2015;85:397–408. 10.1037/ort0000086 26460700

[25] Howell FM. Resistance and gendered racism: middle-class Black women’s experiences navigating reproductive health care systems. Psychol Women Q. 2023;47:494–509.

[26] Rosenthal L, Lobel M. Gendered racism and the sexual and reproductive health of Black and Latina women. Ethn Health. 2020;25:367–92. 10.1080/13557858.2018.1439896 29447448

[27] Taylor JK. Structural racism and maternal health among Black women. J Law Med Ethics. 2020;48:506–17. 10.1177/1073110520958875 33021163

[28] Sakala C, Declercq ER, Turon JM, Corry MP. Listening to mothers in California: a population-based survey of women’s childbearing experiences, full survey report [Internet]. Washington, D.C.: National Partnership for Women & Families.; 2018. Available from: https://www.chcf.org/wp-content/uploads/2018/09/ListeningMothersCAFullSurveyReport2018.pdf

[29] Vedam S, Stoll K, Taiwo TK, Rubashkin N, Cheyney M, Strauss N, et al. The Giving Voice to Mothers study: inequity and mistreatment during pregnancy and childbirth in the United States. Reprod Health [Internet]. 2019 [cited 2024 May 9];16. Available from: https://www.proquest.com/docview/2243235584/abstract/8EEBE04AEC8E4DC4PQ/1 10.1186/s12978-019-0729-2 PMC6558766 31182118

[30] Institute for Gender and Culture. The sexual abuse to maternal mortality pipeline [Internet]. Black Women’s Blueprint; 2019. Available from: https://vawnet.org/material/sexual-abuse-maternal-mortality-pipeline

[31] Gillette-Pierce KT, Richards-McDonald L, Arscott J, Josiah N, Duroseau B, Jacques K, et al. Factors influencing intrapartum health outcomes among Black birthing persons: a discursive paper. J Adv Nurs. 2023;79:1735–44. 10.1111/jan.15520 36461641

[32] Greenfield M, Darwin Z. Trans and non-binary pregnancy, traumatic birth, and perinatal mental health: a scoping review. Int J Transgender Health. 2021;22:203–16. 10.1080/26895269.2020.1841057 PMC8040683 34806082

[33] Lett E, Dowshen NL, Baker KE. Intersectionality and health inequities for gender minority Blacks in the U.S. Am J Prev Med. 2020;59:639–47. 10.1016/j.amepre.2020.04.013 PMC7577994 32792281

[34] Pease MV, Kang N, Oluwakemi G, Jin L, Bradshaw B, Le TP. “It’s like having a superpower”: reclaiming creativity and the intersectional experiences of trans young adults of color. J Couns Psychol. 2024;71:215–28. 10.1037/cou0000737 38829362

[35] Sostre JP, Abreu RL, Vincent D, Lockett GM, Mosley DV. Young Black trans and gender-diverse activists’ well-being among anti-Black racism and cissexism during the COVID-19 pandemic. Psychol Sex Orientat Gend Divers [Internet]. 2023; Available from: https://search.ebscohost.com/login.aspx?direct=true&AuthType=shib&db=psyh&AN=2023-64765-001&authtype=shib&site=ehost-live&scope=site&custid=s5822979

[36] Stroumsa D, Moniz MH, Crissman H, Dalton VK, Tilea A, Pfeiffer PN, et al. Pregnancy outcomes in a US cohort of transgender people. JAMA. 2023;329:1879–81. 10.1001/jama.2023.7688 PMC10176175 37166818

[37] Kotit S, Yacoub M. Cardiovascular adverse events in pregnancy: a global perspective. Glob Cardiol Sci Pract. 2021. 10.21542/gcsp.2021.5. PMC8133785 34036091

[38] Wisner KL, Murphy C, Thomas MM. Prioritizing maternal mental health in addressing morbidity and mortality. JAMA Psychiatr. 2024;81:521–6. 10.1001/jamapsychiatry.2023.5648 38381408

[39] Admon LK, Winkelman TNA, Heisler M, Dalton VK. Obstetric outcomes and delivery-related health care utilization and costs among pregnant women with multiple chronic conditions. Prev Chronic Dis. 2018;15:E21. 10.5888/pcd15.170397 PMC5814150 29420168

[40] Brown CC, Adams CE, George KE, Moore JE. Mental health conditions increase severe maternal morbidity by 50 percent and cost $102 million yearly in the United States. Health Aff Proj Hope. 2021;40:1575–84. 10.1377/hlthaff.2021.00759 PMC8759410 34606352

[41] Geronimus AT, Hicken M, Keene D, Bound J. “Weathering” and age patterns of allostatic load scores among Blacks and Whites in the United States. Am J Public Health. 2006;96:826–33. 10.2105/AJPH.2004.060749 PMC1470581 16380565

[42] Metcalfe A, Wick J, Ronksley P. Racial disparities in comorbidity and severe maternal morbidity/mortality in the United States: an analysis of temporal trends. Acta Obstet Gynecol Scand. 2018;97:89–96. 10.1111/aogs.13245 29030982

[43] Olson DM, Severson EM, Verstraeten BSE, Ng JWY, McCreary JK, Metz GAS. Allostatic load and preterm birth. Int J Mol Sci. 2015;16:29856–74. 10.3390/ijms161226209 PMC4691152 26694355

[44] Riggan KA, Gilbert A, Allyse MA. Acknowledging and addressing allostatic load in pregnancy care. J Racial Ethn Health Disparities. 2021;8:69–79. 10.1007/s40615-020-00757-z PMC7647942 32383045

[45] Coussons-Read ME. Effects of prenatal stress on pregnancy and human development: mechanisms and pathways. Obstet Med. 2013;6:52. 10.1177/1753495X12473751 PMC5052760 27757157

[46] Lawrence B, Kheyfets A, Carvalho K, Dhaurali S, Kiani M, Moky A, et al. The impact of psychosocial stress on maternal health outcomes: a multi-state PRAMS 8 (2016–2018) analysis. J Health Disparities Res Pract [Internet]. 2022;15. Available from: https://digitalscholarship.unlv.edu/jhdrp/vol15/iss2/7

[47] Neiger R. Long-term effects of pregnancy complications on maternal health: a review. J Clin Med. 2017;6:76. 10.3390/jcm6080076 PMC5575578 28749442

[48] Parikh NI, Gonzalez JM, Anderson CAM, Judd SE, Rexrode KM, Hlatky MA, et al. Adverse pregnancy outcomes and cardiovascular disease risk: unique opportunities for cardiovascular disease prevention in women. Circulation. 2021;143:e902–16. 10.1161/CIR.0000000000000961 33779213

[49] Stuart JJ, Tanz LJ, Rimm EB, Spiegelman D, Missmer SA, Mukamal KJ, et al. Cardiovascular risk factors mediate the long-term maternal risk associated with hypertensive disorders of pregnancy. J Am Coll Cardiol. 2022;79:1901–13. 10.1016/j.jacc.2022.03.335 PMC9176211 35550687

[50] Lloyd C. Black adults disproportionately experience microaggressions [Internet]. Gallup.com. 2020 [cited 2024 Dec 5]. Available from: https://news.gallup.com/poll/315695/black-adults-disproportionately-experience-microaggressions.aspx

[51] Jacob G, Faber SC, Faber N, Bartlett A, Ouimet AJ, Williams MT. A systematic review of Black people coping with racism: approaches, analysis, and empowerment. Perspect Psychol Sci. 2023;18:392–415. 10.1177/17456916221100509 PMC10018067 36006823

[52] Jones MS, Womack V, Jérémie-Brink G, Dickens DD. Gendered racism and mental health among young adult U.S. Black women: the moderating roles of gendered racial identity centrality and identity shifting. Sex Roles. 2021;85:221–31.

[53] Shorter-Gooden K. Multiple resistance strategies: how African American women cope with racism and sexism. J Black Psychol. 2004;30:406–25.

[54] Spates K, Evans NM, Watts BC, Abubakar N, James T. Keeping ourselves sane: a qualitative exploration of Black women’s coping strategies for gendered racism. Sex Roles. 2020;82:513–24.

[55] Thomas AJ, Witherspoon KM, Speight SL. Gendered racism, psychological distress, and coping styles of African American women: Cultural Diversity and Ethnic Minority Psychology. Cultur Divers Ethnic Minor Psychol. 2008;14:307–14. 10.1037/1099-9809.14.4.307 18954166

[56] French BH, Lewis JA, Mosley DV, Adames HY, Chavez-Dueñas NY, Chen GA, et al. Toward a psychological framework of radical healing in communities of color. Couns Psychol. 2020;48:14–46.

[57] Adames HY, Chavez-Dueñas NY, Lewis JA, Neville HA, French BH, Chen GA, et al. Radical healing in psychotherapy: addressing the wounds of racism-related stress and trauma. Psychotherapy. 2023;60:39–50. 10.1037/pst0000435 35266783

[58] Implementing radical healing strategies to promote health equity [Internet]. 2022 [cited 2024 Aug 16]. Available from: https://www.nimh.nih.gov/news/media/2022/directors-innovation-speaker-series-implementing-radical-healing-strategies-to-promote-health-equity

[59] Moody AT, Lewis JA, Owens GP. Gendered racism, coping, and traumatic stress among Black women: the moderating roles of the strong black woman schema and womanist attitudes. Psychol Women Q. 2023;47:197–212.

[60] Prilleltensky I. Understanding, resisting, and overcoming oppression: toward psychopolitical validity. Am J Community Psychol. 2003;31:195–201. 10.1023/a:1023043108210 12741700

[61] Jean PL, Mosley DV, Bridges B, Badio K. Thank you black feminism: ten strategies to foster radical healing (for healers and those in need of healing). Women Ther. 2023;46:407–27.

[62] Mosley DV, Neville HA, Chavez-Dueñas NY, Adames HY, Lewis JA, French BH. Radical hope in revolting times: proposing a culturally relevant psychological framework. Soc Personal Psychol Compass. 2020;14:e12512.

[63] Bryant J, Bolt R, Botfield JR, Martin K, Doyle M, Murphy D, et al. Beyond deficit: ‘strengths-based approaches’ in Indigenous health research. Sociol Health Illn. 2021;43:1405–21. 10.1111/1467-9566.13311 34145599

[64] Sukhera J. Narrative reviews: flexible, rigorous, and practical. J Grad Med Educ. 2022;14(4):414–7. 10.4300/JGME-D-22-00480.1 PMC9380636 35991099

[65] Grant MJ, Booth A. A typology of reviews: an analysis of 14 review types and associated methodologies. Health Inf Libr J. 2009;26:91–108. 10.1111/j.1471-1842.2009.00848.x 19490148

[66] Comrie JW, Landor AM, Riley KT, Williamson JD. Anti-Blackness/colorism [Internet]. Boston University Center for Antiracist Research; 2022 May. Available from: https://www.bu.edu/antiracism-center/policy/ antibigotry-convening/

[67] Mollard E, Elya A, Gaines C, Salahshurian E, Riordan E, Moore T, et al. Reclaiming narratives of empowerment around Black maternal health: a strengths-based, community-informed focus group study. Ethn Health. 2024;0:1–17. 10.1080/13557858.2024.2359384 PMC11272425 38805258

[68] Mollard E, Cooper Owens D, Bach C, Gaines C, Maloney S, Moore T, et al. Protective assets reinforced with integrated care and technology (PARITY): protocol for a randomized controlled trial. JMIR Res Protoc. 2024;13:e58580. 10.2196/58580 PMC11342005 39116423

[69] Office of Disease Prevention and Health Promotion. Increase the proportion of pregnant women who receive early and adequate prenatal care [Internet]. Healthy People 2030. [cited 2025 Mar 16]. Available from: https://odphp.health.gov/healthypeople/objectives-and-data/browse-objectives/pregnancy-and-childbirth/increase-proportion-pregnant-women-who-receive-early-and-adequate-prenatal-care-mich-08

[70] Jackson FM, Saran AR, Ricks S, Essien J, Klein K, Roberts D, et al. Save 100 babies©: engaging communities for just and equitable birth outcomes through photovoice and appreciative inquiry. Matern Child Health J. 2014;18:1786–94. 10.1007/s10995-014-1436-9 24474593

[71] Hunte R, Klawetter S, Paul S. “Black nurses in the home is working”: advocacy, naming, and processing racism to improve Black maternal and infant health. Matern Child Health J. 2022;26:933–40. 10.1007/s10995-021-03283-4 PMC10027493 34817758

[72] Duncan R, Coleman J, Herring S, Kawan M, Santoro C, Atre M, et al. Breastfeeding awareness and empowerment (BAE): a Black women-led approach to promoting a multigenerational culture of health. Soc Sci. 2022;12:28. 10.3390/soc12010028 PMC9499340 36160938

[73] Robinson A, Davis M, Hall J, Lauckner C, Anderson AK. It takes an e-village: supporting African American mothers in sustaining breastfeeding through Facebook communities. J Hum Lact. 2019;35:569–82. 10.1177/0890334419831652 30889373

[74] McFarlane SJ, Wright KO, Acheampong B, Francis DB, Callands T, Swartzendruber A, et al. Reframing the experience of childbirth: Black doula communication strategies and client responses during delivery hospitalization. Soc Sci Med. 2024;351:116981. 10.1016/j.socscimed.2024.116981 38781745

[75] OjiNjideka Hemphill N, Crooks N, Zhang W, Fitter F, Erbe K, Rutherford JN, et al. Obstetric experiences of young Black mothers: an intersectional perspective. Soc Sci Med. 2023;317:115604. 10.1016/j.socscimed.2022.115604 PMC9854070 36549014

[76] Woods Barr AL, Austin DA, Smith JL, Schafer EJ. “…[T]his is what we are missing”: the value of communicating infant feeding information across three generations of African American women. J Hum Lact. 2021;37:279–88. 10.1177/0890334421995078 PMC8120634 33632016

[77] Matthews K, Morgan I, Davis K, Estriplet T, Perez S, Crear-Perry JA. Pathways to equitable and antiracist maternal mental health care: insights from Black women stakeholders. Health Aff (Millwood). 2021;40(1597–7):1A-6A. 10.1377/hlthaff.2021.00808 34606342

[78] Gregorio VR, Mantri S. Portals to the past and bridges to the future: exploring the impact of doulas on the birthing experiences of Black and Latinx women. Med Humanit. 2024;50:306–11. 10.1136/medhum-2023-012840 38604656

[79] Rubin SE, Hines J. “As long as i got a breath in my body”: risk and resistance in Black maternal embodiment. Cult Med Psychiatry. 2023;47:495–518. 10.1007/s11013-022-09780-7 35381902

[80] Mannor KM, Malcoe LH. Uses of theory in racial health disparities research: a scoping review and application of public health critical race praxis. Ann Epidemiol. 2022;66:56–64. 10.1016/j.annepidem.2021.11.007 34793963

[81] Buchanan NT, Perez M, Prinstein MJ, Thurston IB. Upending racism in psychological science: strategies to change how science is conducted, reported, reviewed, and disseminated. Am Psychol. 2021;76:1097–112. 10.1037/amp0000905 34990171

[82] Sherman ADF, Febres-Cordero S, Bonds Johnson K, Clark KD, Klepper M, Duroseau B, et al. Intersectionality in nursing research: a scoping review. Int J Nurs Stud Adv. 2023;5:100155. 10.1016/j.ijnsa.2023.100155 PMC10655863 37982092

[83] Shin RQ, Welch JC, Kaya AE, Yeung JG, Obana C, Sharma R, et al. The intersectionality framework and identity intersections in the Journal of Counseling Psychology and the Counseling Psychologist: a content analysis. J Couns Psychol. 2017;64(5):458–74. 10.1037/cou0000204 29048193

[84] Roberts SO, Bareket-Shavit C, Dollins FA, Goldie PD, Mortenson E. Racial inequality in psychological research: trends of the past and recommendations for the future. Perspect Psychol Sci. 2020;15:1295–309. 10.1177/1745691620927709 32578504

[85] Auguste E, Bowdring M, Kasparek SW, McPhee J, Tabachnick AR, Tung I, et al. Psychology’s contributions to anti-Blackness in the United States within psychological research, criminal justice, and mental health. Perspect Psychol Sci. 2023;18:1282–305. 10.1177/17456916221141374 PMC10715736 36753574

[86] Baumann AA, Shelton RC, Kumanyika S, Haire-Joshu D. Advancing healthcare equity through dissemination and implementation science. Health Serv Res. 2023;58(S3):327–44. 10.1111/1475-6773.14175 PMC10684051 37219339

[87] Owens DC, Fett SM. Black maternal and infant health: historical legacies of slavery. Am J Public Health. 2019;109:1342–5. 10.2105/AJPH.2019.305243 PMC6727302 31415204

[88] Chen CY, Kahanamoku SS, Tripati A, Alegado RA, Morris VR, Andrade K, et al. Systemic racial disparities in funding rates at the National Science Foundation. Rodgers P, Gilpin N, Bernard R, editors. eLife. 2022;11:e83071. 10.7554/eLife.83071 PMC9708090 36444975

[89] Abrams J, Tabaac A, Jung S, Else-Quest N. Considerations for employing intersectionality in qualitative health research. Soc Sci Med. 1982;2020(258):113138. 10.1016/j.socscimed.2020.113138 PMC7363589 32574889

[90] Brown AF, Ma GX, Miranda J, Eng E, Castille D, Brockie T, et al. Structural interventions to reduce and eliminate health disparities. Am J Public Health. 2019;109:S72–8. 10.2105/AJPH.2018.304844 PMC6356131 30699019

[91] Firoz T, McCaw-Binns A, Filippi V, Magee LA, Costa ML, Cecatti JG, et al. A framework for healthcare interventions to address maternal morbidity. Int J Gynaecol Obstet. 2018;141:61–8. 10.1002/ijgo.12469 PMC6001624 29851114

[92] Katon JG, Enquobahrie DA, Jacobsen K, Zephyrin LC. Policies for reducing maternal morbidity and mortality and enhancing equity in maternal health [Internet]. Commonw. Fund. 2021 [cited 2025 Feb 6]. Available from: https://www.commonwealthfund.org/publications/fund-reports/2021/nov/policies-reducing-maternal-morbidity-mortality-enhancing-equity

[93] Toval CA, Darivemula SM, Wilson TD, Conklin JL, Young OM. Interventions to mitigate pregnancy-related mortality and morbidity in Black birthing people: a systematic review. Am J Obstet Gynecol MFM. 2024;6:101464. 10.1016/j.ajogmf.2024.101464 39147362

[94] Causadias JM, Vitriol JA, Atkin AL. Do we overemphasize the role of culture in the behavior of racial/ethnic minorities? Evidence of a cultural (mis)attribution bias in American psychology. Am Psychol. 2018;73:243–55. 10.1037/amp0000099 29355353

[95] Beames JR, Kikas K, O’Gradey-Lee M, Gale N, Werner-Seidler A, Boydell KM, et al. A new normal: integrating lived experience into scientific data syntheses. Front Psychiatry. 2021;12:763005. 10.3389/fpsyt.2021.763005 PMC8585932 34777064

[96] Crear-Perry JA. Black mothers’ health: how upending the typical grant funding structure is shifting power to improve it. Health Aff Forefr [Internet]. 2019 [cited 2025 Feb 6]; Available from: https://www.healthaffairs.org/do/10.1377/forefront.20190605.951106/full/

[97] Dankwa-Mullan I, Rhee KB, Stoff DM, Pohlhaus JR, Sy FS, Stinson N, et al. Moving toward paradigm-shifting research in health disparities through translational, transformational, and transdisciplinary approaches. Am J Public Health. 2010;100:S19-24. 10.2105/AJPH.2009.189167 PMC2837422 20147662

[98] Scott KA, Bray S, McLemore MR. First, do no harm: why philanthropy needs to re-examine its role in reproductive equity and racial justice. Health Equity. 2020;4:17–22. 10.1089/heq.2019.0094 PMC7097698 32219193

[99] Agénor M, Zubizarreta D, Geffen S, Ramanayake N, Giraldo S, McGuirk A, et al. “Making a way out of no way:” understanding the sexual and reproductive health care experiences of transmasculine young adults of color in the United States. Qual Health Res. 2022;32:121–34. 10.1177/10497323211050051 PMC10921419 34851198

[100] Misiolek BA, Batra P, Hicks L, Tupper C, Lewis NJW. Engaging Black and Latine transgender and gender diverse persons in research: Advice from a community-based focus group. RTI Press [Internet]. 2024 [cited 2024 Nov 2]; Available from: https://www.rti.org/rti-press-publication/engaging-black-latine-transgender-gender-diverse-persons-research-advice-community-based-focus-group

[101] Rivera AV, Lopez JM, Braunstein SL. Recruiting Black and Latina/Hispanic transgender women for HIV research: implementation of respondent-driven sampling and factors associated with peer recruitment. Transgend Health. 2024;9:339–47. 10.1089/trgh.2021.0201 PMC11456766 39385953

[102] Hoppe TA, Litovitz A, Willis KA, Meseroll RA, Perkins MJ, Hutchins BI, et al. Topic choice contributes to the lower rate of NIH awards to African-American/Black scientists. Sci Adv. 2019;5:eaaw7238. 10.1126/sciadv.aaw7238 PMC6785250 31633016

[103] Kozlowski D, Larivière V, Sugimoto CR, Monroe-White T. Intersectional inequalities in science. Proc Natl Acad Sci U S A. 2022;119:e2113067119. 10.1073/pnas.2113067119 PMC8764684 34983876

[104] Loui M, Fiala SC. Inequities in academic publishing: where is the evidence and what can be done? Am J Public Health. 2024;114:377–81. 10.2105/AJPH.2024.307587 PMC10937608 38478868

[105] Salazar JW, Claytor JD, Habib AR, Guduguntla V, Redberg RF. Gender, race, ethnicity, and sexual orientation of editors at leading medical and scientific journals: a cross-sectional survey. JAMA Intern Med. 2021;181:1248–51. 10.1001/jamainternmed.2021.2363 PMC8424470 34115835

[106] Adams J, Hillier-Brown FC, Moore HJ, Lake AA, Araujo-Soares V, White M, et al. Searching and synthesising ‘grey literature’ and ‘grey information’ in public health: critical reflections on three case studies. Syst Rev. 2016;5:164. 10.1186/s13643-016-0337-y PMC5041336 27686611

[107] Christensen MC, Todić J, McMahon SM. Bridging the grey gap: conducting grey literature reviews for ethical social work practice and research. J Soc Soc Work Res. 2022;13:609–35.

[108] Livne-Tarandach R, Steckler E, Leigh J, Wheeler-Smith S. Cultivating organizations as healing spaces: a typology for responding to suffering and advancing social justice. Hum Manag J. 2021;6:373–404. 10.1007/s41463-021-00112-2 PMC8664364 40477506

[109] Cowan ES, Dill LJ, Sutton S. Collective healing: a framework for building transformative collaborations in public health. Health Promot Pract. 2022;23(3):356–60. 10.1177/15248399211032607 34549646

[110] John Hopkins Medicine. Four common pregnancy complications [Internet]. 2024 [cited 2025 Mar 6]. Available from: https://www.hopkinsmedicine.org/health/conditions-and-diseases/staying-healthy-during-pregnancy/4-common-pregnancy-complications

[111] Davis S, O’Brien A-M. Let’s talk about racism: strategies for building structural competency in nursing. Acad Med. 2020;95:S58. 10.1097/ACM.0000000000003688 32889918

[112] Metzl JM, Hansen H. Structural competency: Theorizing a new medical engagement with stigma and inequality. Soc Sci Med. 1982;2014(103):126–33. 10.1016/j.socscimed.2013.06.032 PMC4269606 24507917

[113] Melino K. Structural competency in health care. Nurs Clin North Am. 2022;57:433–41. 10.1016/j.cnur.2022.04.009 PMC9300050 35985730

[114] Diouf F, Thompson T, Silesky M, Bonnevie E. A call to action: supporting Black maternal and infant health using the collective impact model. Matern Child Health J. 2024;28:1265–71. 10.1007/s10995-024-03937-z 38844649

[115] Greenwood BN, Hardeman RR, Huang L, Sojourner A. Physician–patient racial concordance and disparities in birthing mortality for newborns. Proc Natl Acad Sci U S A. 2020;117:21194–200. 10.1073/pnas.1913405117 PMC7474610 32817561

[116] Snyder JE, Upton RD, Hassett TC, Lee H, Nouri Z, Dill M. Black representation in the primary care physician workforce and its association with population life expectancy and mortality rates in the US. JAMA Netw Open. 2023;6:e236687. 10.1001/jamanetworkopen.2023.6687 PMC10105312 37058307

[117] Takeshita J, Wang S, Loren AW, Mitra N, Shults J, Shin DB, et al. Association of racial/ethnic and gender concordance between patients and physicians with patient experience ratings. JAMA Netw Open. 2020;3:e2024583. 10.1001/jamanetworkopen.2020.24583 PMC7653497 33165609

[118] Black Mamas Matter Alliance, Center for Reproductive Rights. Black Mamas Matter: advancing the human right to safe and respectful maternal health care [Internet]. 2018. Available from: http://blackmamasmatter.org/wp-content/uploads/2018/05/USPA_BMMA_Toolkit_Booklet-Final-Update_Web-Pages-1.pdf

[119] National Partnership for Women and Families. Black women’s maternal health: a multifaceted approach to addressing persistent and dire health inequities [Internet]. 2023 Dec. Available from: https://nationalpartnership.org/wp-content/uploads/black-womens-maternal-health.pdf

[120] Mollard E, Cottrell C. Maternal adaptive capacity: a strengths-based theory to guide maternal health research. J Midwifery Womens Health. 2023;68:376–82. 10.1111/jmwh.13485 37010188

